# Genes involved in the regulation of different types of autophagy and their participation in cancer pathogenesis

**DOI:** 10.18632/oncotarget.26126

**Published:** 2018-09-28

**Authors:** Martyna Bednarczyk, Nikola Zmarzły, Beniamin Grabarek, Urszula Mazurek, Małgorzata Muc-Wierzgoń

**Affiliations:** ^1^ Department of Internal Diseases, School of Public Health in Bytom, Medical University of Silesia in Katowice, 40–055 Katowice, Poland; ^2^ Department of Molecular Biology, School of Pharmacy with The Division of Laboratory Medicine in Sosnowiec, Medical University of Silesia in Katowice, 40–055 Katowice, Poland

**Keywords:** autophagy, lysosome, chaperones, mitochondria, cancer

## Abstract

Autophagy is a highly conserved mechanism of self-digestion that removes damaged organelles and proteins from cells. Depending on the way the protein is delivered to the lysosome, four basic types of autophagy can be distinguished: macroautophagy, selective autophagy, chaperone-mediated autophagy and microautophagy. Macroautophagy involves formation of autophagosomes and is controlled by specific autophagy-related genes. The steps in macroautophagy are initiation, phagophore elongation, autophagosome maturation, autophagosome fusion with the lysosome, and proteolytic degradation of the contents. Selective autophagy is macroautophagy of a specific cellular component. This work focuses on mitophagy (selective autophagy of abnormal and damaged mitochondria), in which the main participating protein is PINK1 (phosphatase and tensin homolog-induced putative kinase 1). In chaperone-mediated autophagy, the substrate is bound to a heat shock protein 70 chaperone before it is delivered to the lysosome. The least characterized type of autophagy is microautophagy, which is the degradation of very small molecules without participation of an autophagosome. Autophagy can promote or inhibit tumor development, depending on the severity of the disease, the type of cancer, and the age of the patient. This paper describes the molecular basis of the different types of autophagy and their importance in cancer pathogenesis.

## INTRODUCTION

Autophagy has two basic functions in the cell. On the one hand, it is a mechanism for removing damaged cellular components or organelles by self-digestion. On the other hand, as a catabolic process, it generates substrates necessary to maintain cellular energy homeostasis when there is limited access to nutrients. Autophagy is also considered to be type II programmed cell death [1, 2, 3]. Cancer cells can acquire resistance to apoptosis (type I programmed cell death) by expressing anti-apoptotic proteins such as B-cell lymphoma 2 (BCL-2) or by downregulating pro-apoptotic proteins. Abnormal apoptosis contributes to cancer induction and chemotherapy resistance, suggesting that diversion to an alternative cell death pathway such as autophagy may allow more beneficial therapeutic effects [4].

Autophagy is activated in response to long-term nutrient deficiency, tissue remodeling, organelle quality control, immune system responses and cellular stress [5]. There are four major intracellular autophagy pathways: macroautophagy, microautophagy, chaperone-mediated autophagy (CMA) and selective autophagy. Selective autophagy is specific for the substrate, e.g., for mitochondria - mitophagy, lipids - lipophagy, pathogens - xenophagy, peroxisomes - pexophagy. The classification of these types of autophagy is based on the size of the eliminated substrates and the scale of their degradation. The different types of autophagy share the common feature of the lysosomal degradation of damaged proteins, but differ in their mechanisms of delivering the substrate to the lysosome [2]. Table 1 presents the differences and similarities among the four types of autophagy. This paper focuses on the genes regulating the different types of autophagy and their participation in cancer pathogenesis.

**Table 1:** Comparison of autophagy types

	Macroautophagy	Selective autophagy - mitophagy	Chaperone-mediated autophagy	Microautophagy
Autophagosome formation	Formed	Formed	Not formed	Not formed
Degradation of substrate in lysosome	Yes	Yes	Yes	Yes
Key proteins regulating the process	BECN1, proteins from the ATG family	PINK1	LAMP2	Not identified
Process selectivity	Non-specific process	Mitophagy - specific for mitochondria	Applies only to substrates containing a KFERQ sequence	Process specific to very small organelles

## MACROAUTOPHAGY

Macroautophagy occurs in all eukaryotic organisms and is the main degradation system of intracellular components. It is induced, among other things, by nutrient deficiencies, hypoxia and oxidative stress. Macroautophagy also prevents the accumulation of cytotoxic components in the cell. Although autophagy was identified in mammalian cells about 50 years ago, the molecular basis of this process has only been understood in the last decade [6]. Studies of signaling pathways in yeast greatly contributed to the development of the mammalian autophagy mechanism [7]. About 30 genes from the autophagy-related (ATG) family regulate the autophagy process. These genes were first identified in yeast, and then their orthologues were identified in humans [2, 6, 8].

**Figure 1:**
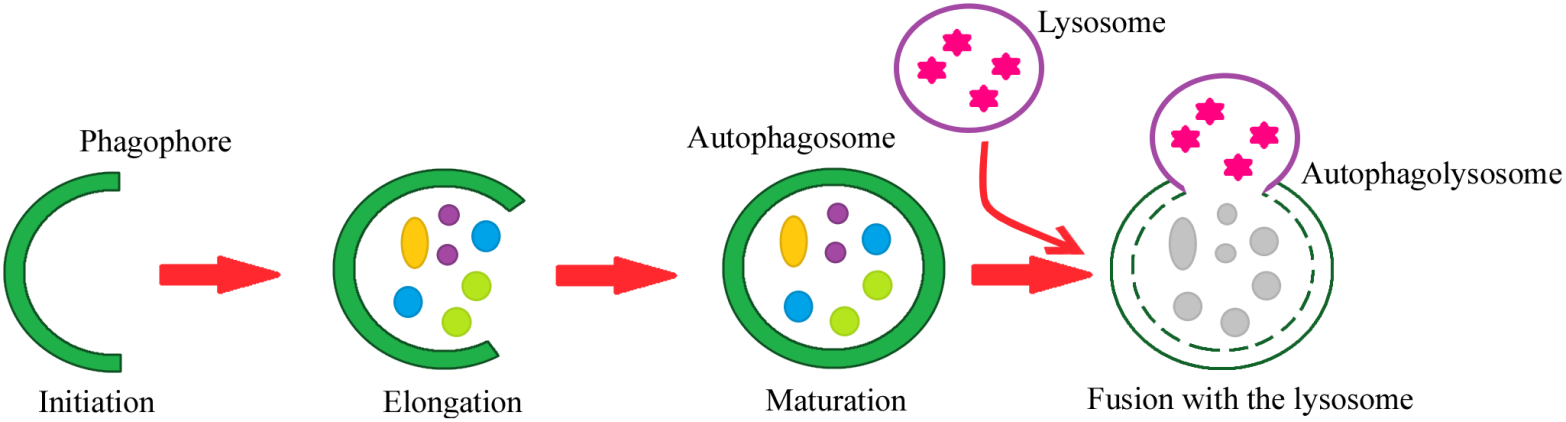
Steps of the autophagy process Phagophore – a double membrane that encloses and isolates the cytoplasmic components during macroautophagy. Autophagosome – a spherical structure with a double membrane. It is the key structure in macroautophagy, the intracellular degradation system for cytoplasmic contents. Autophagolysosome – the structure created by the fusion of the autophagosome with the lysosome. Lysosome – the membrane-enclosed organelle that contains an array of enzymes capable of breaking down substrates.

Autophagy can be divided into the following steps: initiation, phagophore elongation, autophagosome maturation, autophagosome fusion with the lysosome, and proteolytic degradation of the contents. The process begins with the formation of a crescent-shaped double membrane called the phagophore or isolation membrane, in which the damaged proteins are enclosed. The phagophore forms as a result of the fusion of multiple vesicles derived from the endoplasmic reticulum [2, 9, 10, 11]. The next step is phagophore elongation. In the final phase of the process, the outer membrane of the autophagosome fuses with the lysosome, forming an autolysosome. Under the influence of lysosomal enzymes, the inner membrane of the autolysosome is digested, including its contents [2, 11, 12, 13]. Figure 1 depicts the steps of macroautophagy. About 18 ATG proteins (which were first identified in yeast) participate in this process. In addition, two ubiquitin-conjugation systems are involved – ATG8-PE and ATG5-ATG12-ATG16 – which affect, among other things, the formation and size of the autophagosome [14, 15].

**Figure 2:**
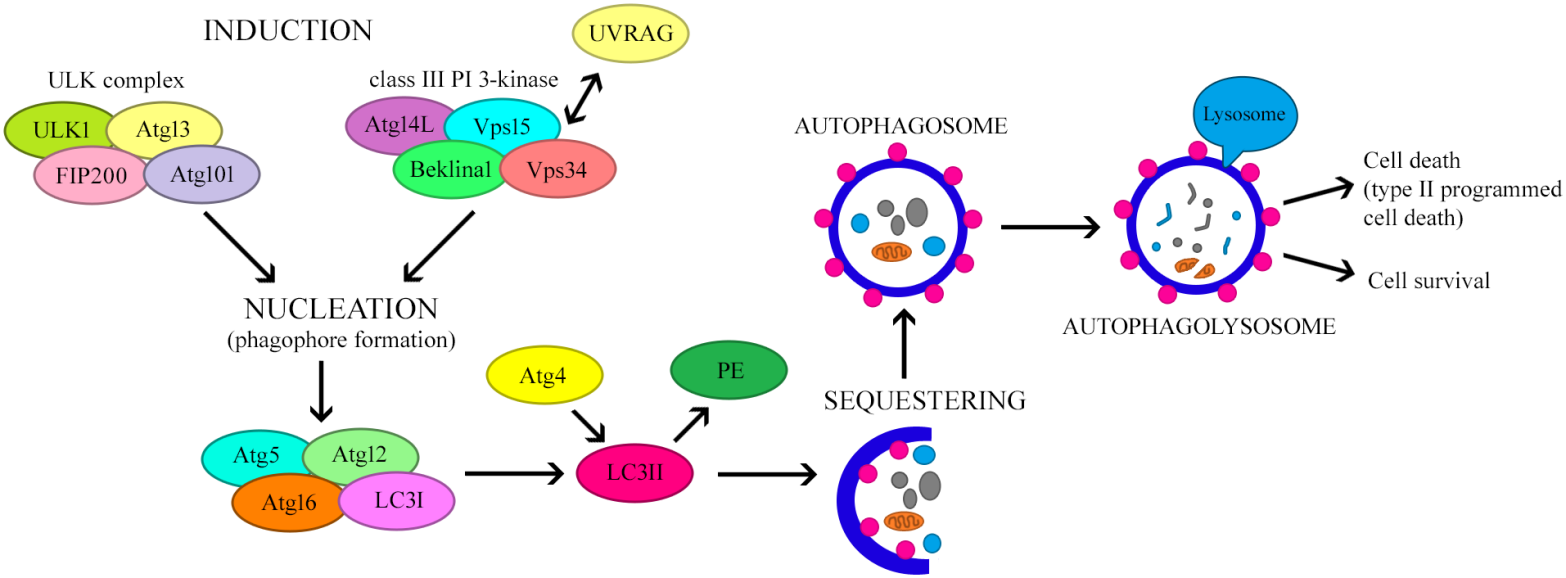
The mechanism regulating autophagy

Amino acid deficiency is one of the most potent inducers of autophagy. Moreover, insulin and growth factors may influence this process [15, 16]. The initiation of autophagosome formation involves two major complexes that require the participation of other proteins (Figure 2). The first complex contains class III phosphoinositide 3-kinase (PI3K-III), Beclin-1 and p150 serine kinase. The second complex consists of UNC-51-like autophagy activating kinase 1 (ULK1), a serine/threonine kinase. ULK1 is active in the presence of other proteins associated with autophagy: ULK2, focal adhesion kinase family interacting protein of 200 kDa (FIP200), mATG13 and ATG101. ATG101 is present in the phagophore and is responsible for phosphorylating mATG13 and ULK1, whereas FIP200 stabilizes and phosphorylates ULK1 [14, 17, 18]. The first of these complexes participates in the early phase of autophagosome formation [19].

Under normal growth conditions, the mammalian target of rapamycin (mTOR) complex inhibits the formation of the ULK complex, thereby inhibiting autophagy and inducing the dissociation of the components of ULK. However, certain stimuli, including hunger and hypoxia, inhibit mTOR by activating the ULK complex, thus promoting the formation of the isolation membrane or phagophore [20]. During nutrient deficiency, the rate of pre-autophagosome formation increases [7]. In the next step, which is nucleation, PI3K-III bind to Beclin-1, a key regulator of autophagy. On the other hand, Beclin-1 may be inhibited by the anti-apoptotic proteins BCL-2 and BCL-extra large (BCL-XL) [10]. Furthermore, vacuolar protein sorting 34 (VPS34) kinase interacts with Beclin-1, thus increasing the production of phosphatidylinositol triphosphate (PIP3), which is necessary for the elongation of the phagophore and the recruitment of ATG proteins to it [12].

Other proteins associated with the Beclin complex (e.g., activating molecule in BECN1-regulated autophagy [AMBRA1] and ultraviolet irradiation resistance-associated gene [UVRAG]) may also influence its functions [21]. UVRAG participates in at least four mechanisms that regulate autophagy. Firstly, ATG14L competes with UVRAG for binding to the Beclin-1 complex. Secondly, UVRAG interacts with BAX-interacting factor 1, an essential protein for autophagy. Thirdly, UVRAG interacts with the class C VPS/HOPS proteins, inducing the fusion of the autophagosome with the late endosome/lysosome. Fourthly, the recently identified Rubicon protein combines with UVRAG-Beclin-1-hVPS34-p150, inhibiting the maturation of the autophagosome [2].

The elongation and maturation of the autophagosome involves ubiquitin-like complexes such as ATG12-ATG5 and microtubule-associated protein light chain 3 (LC3)-II-phosphatidylethanolamine (LC3-II-PE) [22, 23]. In the first case, ATG7 (ubiquitin-activating enzyme E1-like protein) activates ATG12 by covalently binding to its C-terminal glycine residue in an ATP-dependent manner [16, 24]. ATG12 is transferred to ATG10 (ubiquitin-activating enzyme E2-like protein), and then conjugates with ATG5, forming the ATG12-ATG5 complex [24, 25]. ATG12-ATG5 binds non-covalently to ATG16L to form the ATG12-ATG5-ATG16L complex. This leads to multimerization and the creation of a tetramer (ATG12-ATG5-ATG16L), which is necessary for phagophore elongation and autophagosome formation. Recent studies have demonstrated that this complex causes the curvature of the phagophore. During autophagosome formation, ATG16L dissociates from the complex, and therefore cannot be used as a marker of autophagy [22, 26].

The second complex involved in autophagosome formation is LC3-II-PE. LC3 is initially synthesized as a precursor protein, pro-LC3; however, with the participation of ATG, it is then directly processed into the LC3-I form. ATG4, ATG7 and ATG3 attach PE to LC3. This converts the soluble, cytoplasmic form (LC3-I) into a lipophilic form (LC3-II), which is incorporated into both sides of the autophagosomal isolation membrane. After the autophagosome fuses with the lysosome (as described below), LC3-II on the cytoplasmic side is detached by ATG4 and recycled, while LC3-II on the inside of the autophagosome is degraded by lysosomal enzymes in the autolysosome. The concentration of LC3-II correlates with the number of autophagosomes formed, so LC3-II is considered the most reliable marker of autophagy [8, 13, 20, 23, 25].

At the end of phagophore membrane elongation, the resulting autophagosome moves along microtubules to a lysosome-enriched microtubule organizing center. With the participation of SNARE or UVRAG, the autophagosome fuses with the lysosome to form the autolysosome [10]. Presenilin and GTP-associated RAB7 are necessary in this process, as well as cathepsins B and D, which are present inside the lysosome and involved in autolysosome maturation [24].

### The dual role of autophagy in the pathogenesis of cancer

Autophagy is a key contributor to the pathogenesis of many human diseases, including neurodegenerative, infectious, cardiovascular and metabolic diseases, as well as cancer [20].

Until recently, the problem of cell death was considered only in terms of apoptosis and necrosis. Abnormal apoptosis leads to genetic damage and carcinogenesis. Much has been published about the mutations and changes in the expression of genes associated with apoptosis in human cancers, such as FAS or caspases [14].

**Figure 3:**
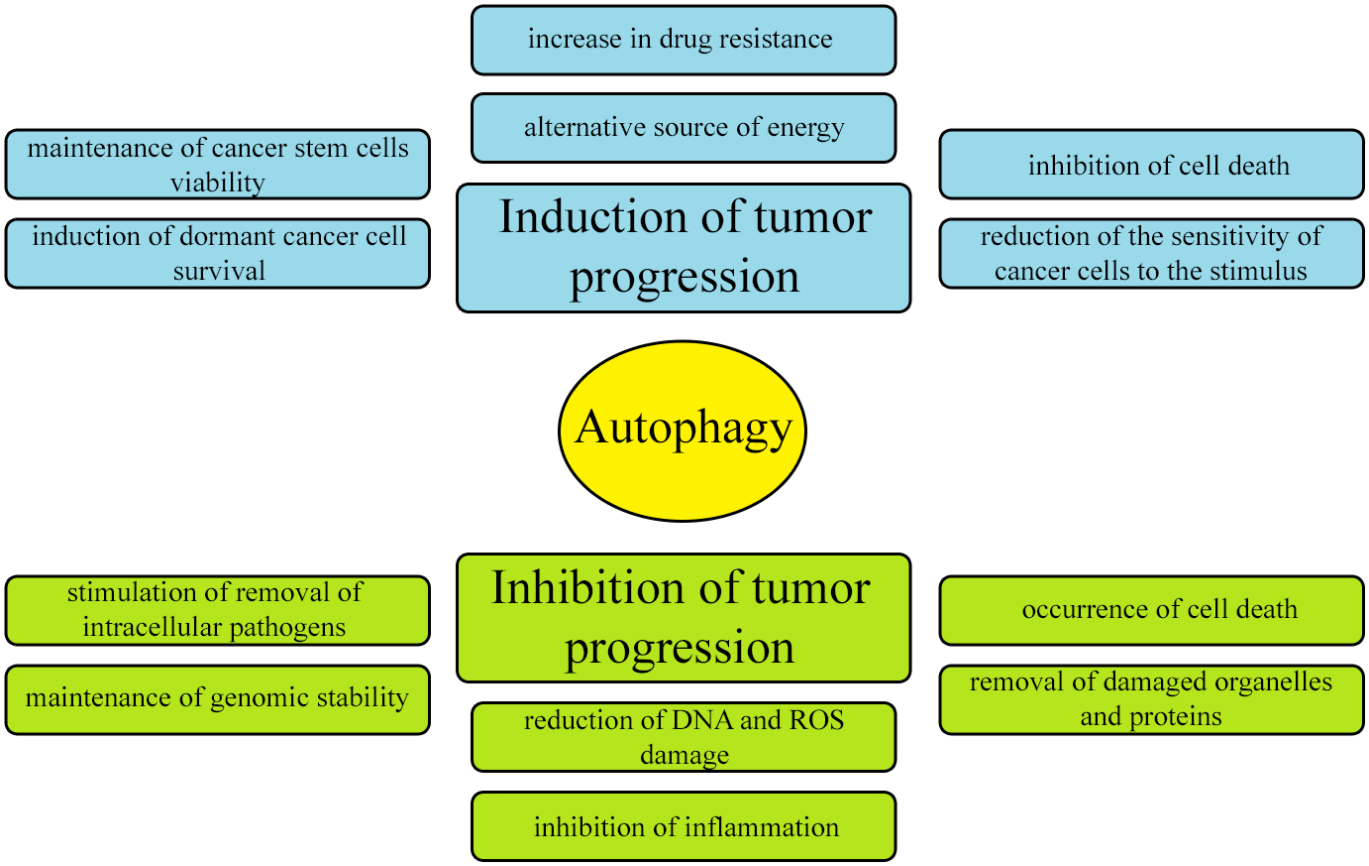
Dual and contradictory roles of autophagy in oncogenesis Autophagy can either inhibit or promote neoplastic transformation.

Literature data provide conflicting information on the involvement of autophagy in tumor pathogenesis. Some reports indicate that autophagy inhibits cancer development, while others suggest that it stimulates the formation of tumors and protects cancer cells from death. The currently accepted hypothesis is that autophagy can either be an enemy or an ally of cancer, depending on the physiological or pathophysiological condition of the cell (Figure 3) [12]. This dual role of autophagy results from the different contexts of its activation. Autophagy occurs at a basal level in all types of tissues, removing damaged organelles and improperly folded proteins, thereby governing the circulation of proteins in the cell and maintaining homeostasis. Thus, under physiological conditions (when nutrient levels are normal), autophagy protects cells from cancer development. In contrast, during nutrient deficiency, hypoxia and bacterial infections, autophagy occurs at a higher level, thus contributing to the cellular adaptation to stress, but also protecting cancer cells from death [12].

Regarding the first context of autophagy (physiological conditions), many studies have indicated that this pathway can prevent the initiation of cancer. Although the mechanisms are not fully understood, the removal of damaged proteins and organelles such as mitochondria in the early stages of the disease is likely to reduce tumor cell growth, mutagenesis and other damage caused by reactive oxygen species (ROS). Accordingly, if the level of autophagy is reduced, the cell loses the ability to remove damaged proteins and organelles and starts to accumulate cytotoxic components that can damage DNA and induce oncogenesis [12]. When primary epithelial cells become immortal, cell death pathways (both apoptosis and autophagy) are selectively inactivated. Apoptosis, necrosis and autophagy have all been shown to remove epithelial cells, so autophagy in the early stages of epithelial cancer could prevent its further development [14, 20].

The gene expression changes in cancer tissue compared to healthy tissue have also indicated the protective role of autophagy. When there are defects in autophagy induction (for instance, due to the monoallelic deletion of Beclin-1), cells are more easily transformed into cancer cells [12]. Indeed, *BECN1* (encoding Beclin-1), which is associated with the formation of the autophagosome, is often deleted in breast and ovarian cancer. In addition, *p53* and phosphatase and tensin homolog *(PTEN)*, which normally induce autophagy, are the most commonly mutated tumor suppressor genes. On the other hand, the oncogenic protein BCL-2, which directly binds to Beclin-1, inhibits autophagy [14, 20].

Regarding the second context of autophagy (pathophysiological conditions), the higher level of autophagy during nutrient deprivation allows the cell to degrade proteins and organelles to acquire amino acids, fatty acids and nucleotides for the synthesis of new macromolecular compounds. Thus, autophagy has the protective function of allowing cell survival during nutrient deficiency. In addition, autophagy promotes DNA repair and reduces mitochondrial disorders. However, autophagy also inhibits apoptosis, so it is assumed that when limited angiogenesis leads to nutrient deprivation and hypoxia, autophagy keeps tumor cells alive. Therefore, it has been hypothesized that an increase in the level of autophagy enhances the growth of solid tumors, while a decrease in autophagy significantly limits tumor growth [14, 20].

In the advanced stages of cancer, this cancer-promoting activity of autophagy is particularly evident [27]. When angiogenesis is very advanced, autophagy provides access to nutrients that are necessary for the metabolism and growth of cancer cells. In addition, autophagy induces resistance to chemotherapy [14, 20]. Autophagy also promotes the survival of p53-deficient cancer cells under conditions of nutrient deficiency or hypoxia [27]. Many preclinical and clinical studies have already been undertaken to develop therapeutic agents directly targeting the autophagy pathway (induction and inhibition), and these agents may be used in the future to treat neoplastic diseases [28].

### Proteins involved in autophagy and carcinogenesis

#### LAMP family

Over 25 lysosomal proteins are known to be involved in processes such as the acidification of the lysosome, the fusion of the membrane and the transport of degradation products to the cytoplasm. Proteins belonging to the lysosome-associated membrane protein (LAMP) family are highly glycosylated transmembrane glycoproteins [29, 30]. Three proteins are included in this group – LAMP1, LAMP2 and LAMP3 – which share the common feature of a Gly-Tyr motif [31].

LAMP1 and LAMP2 constitute 50% of the lysosomal membrane proteins. The function of LAMP2 will be discussed later, due to the participation of this protein in a different type of autophagy. LAMP1 is a type 1 transmembrane protein, and although its function is not fully understood [29], its location in the lysosomal membrane indicates its probable involvement in macroautophagy. LAMP1 is necessary for effective autophagy, and its main tasks are to regulate the mobility of the lysosome and to fuse the endosome/lysosome with the autophagosome [31, 32]. LAMP1 is expressed not only in the membranes of endosomes/lysosomes, but also in the cell membrane. Moreover, LAMP1 is highly active in the membranes of aggressively metastatic tumor cells. In particular, metastatic colon cancer cells are characterized by high levels of *LAMP1* mRNA in relation to cells with low metastatic potential, which may indicate the participation of LAMP1 in cell adhesion and migration [33, 34, 35]. LAMP1 is upregulated in many types of cancer, including colorectal adenocarcinoma. In addition, the transcription of *LAMP1* increases with the degree of cancer advancement, implying that its mRNA levels correlate with malignant tumor transformation. These and other data suggest that LAMP1 is involved in tumor progression, metastasis and invasion [36, 37].

The third LAMP family member, LAMP3, was discovered relatively recently, and is quite similar to LAMP1 and LAMP2 [30, 38]. *LAMP3* is located on chromosome 3q27, a region that is amplified in many types of cancer [39]. This protein is mainly present in the lysosomal membrane [40]. Though its exact function has not yet been determined, LAMP3 probably increases cell survival by participating in macroautophagy and inducing the fusion of the autophagosome with the lysosome, like LAMP1. In contrast to LAMP1 and LAMP2, LAMP3 is only expressed in specific tissues and conditions. Its overexpression has been observed in many types of human cancer, including ovarian, breast, cervical, lung, colorectal, pancreatic and liver cancer. The upregulation of LAMP3 is associated with tumor metastasis and a poor prognosis [29, 37, 38, 41]. LAMP3 is rarely expressed in normal cells [42], but promotes the migration and invasion of cancer cells [43]. The upregulation of *LAMP3* mRNA also correlates with a poor prognosis and resistance to treatment, especially chemotherapy and radiotherapy [42, 44].

### DRAM1 and p53

As described above, autophagy can either induce tumor progression or inhibit the development of the disease. However, the stimuli and signaling pathways that regulate this dual nature of autophagy remain poorly defined. Cancer progression is a multistep process involving alterations in both oncogenes and tumor suppressor genes. The gene encoding the p53 protein, which has both positive and negative effects on autophagy, is the most common target for mutation in human cancer. When p53 is expressed at basal levels in the cytoplasm, it inhibits autophagy. However, in response to cellular stressors such as DNA damage or ribosomal stress, p53 expression increases significantly above the baseline level. As a result, p53 accumulates in the cell nucleus, where it transcriptionally activates a number of genes that inhibit tumor progression [45, 46, 47, 48].

p53 activates many genes that promote autophagy, such as the newly discovered autophagy regulator induced by cellular stress, damage-regulated autophagy modulator 1 (*DRAM1*). In addition, p53 induces autophagy in a DRAM1-dependent manner. As DRAM1 is present in the membrane of the lysosome (the organelle involved in the final phase of autophagy), it is assumed that DRAM1 promotes the fusion of the autophagosome with the lysosome [45, 46, 47, 48]. In addition, since p53 is a tumor suppressor responsible for apoptotic cell death and autophagy induction, it is believed that DRAM1 connects the autophagy pathway with apoptosis [14, 49].

DRAM1 contains a signal peptide that directs it to the endoplasmic reticulum, as well as six hydrophobic transmembrane regions. DRAM1 can occupy different intracellular locations. The exact relationship between the function and subcellular localization of DRAM1 is not fully understood; however, it is thought that DRAM1 present in the mitochondria induces apoptosis through mitophagy [50]. DRAM1 also induces autophagy by stimulating ATPase activity in the vacuoles, and by increasing lysosomal acidification; thus, it is assumed that DRAM1 regulates autophagy partly through the lysosomes [51].

DRAM1 potentially suppresses tumor development, and its mRNA levels are reduced in many types of cancer. The downregulation of *DRAM1* in tumor cells is the result of hypermethylation within CpG islands in its promoter region, as well as other mechanisms, such as the epigenetic modification of core histones near the *DRAM1* gene [48, 52].

### Beclin-1

In 1998, *BECN1* was identified in chromosome 17q21 within a region that is often deleted in breast, ovarian and prostate cancer. Mutations in *BECN1* are often present in various types of tumors, so it is believed that Beclin-1 is a tumor suppressor [53, 28]. Reports on Beclin-1 have focused primarily on its participation in pre-autophagosome formation through its binding to other proteins. Beclin-1 is mainly located in the Golgi apparatus, endoplasmic reticulum and mitochondria. In colon cancer, it has also been found to be localized in the cell nucleus [54].

*BECN1* was the first connection described between autophagy and cancer [55]. In some types of tumors, such as liver and lung cancer, Beclin-1 expression is reduced, indicating that autophagy may inhibit the development of these cancers [28, 56]. Moreover, in tumors of the gastrointestinal tract, increased expression of *BECN1* has been observed in the first stages of the disease, while Beclin-1 activity is reduced in subsequent stages of cancer progression. Higher levels of Beclin-1 may be associated with a better prognosis in patients with colorectal cancer. On the other hand, the deletion of Beclin-1 in hepatocellular carcinoma cells was associated with the recurrence of the disease [54, 56].

Based on the existing reports, it is believed that Beclin-1 has a dual function in oncogenesis. Beclin-1 promotes both the early maturation of endosomes and the activation of the class III PI3-kinase complex (PI3KC3/VPS34). Reduced expression of Beclin-1 inhibits autophagy, which may induce tumor formation by causing oxidative stress, DNA damage or genomic instability. On the other hand, diminished Beclin-1 activity may also delay the early maturation of endosomes, thus increasing the stability of growth factor receptor signaling and contributing to the progression of the neoplastic process. However, further research is needed to determine how the simultaneous reduction in autophagy and increase in signaling influence tumor development and progression [57].

In terms of its structure, Beclin-1 contains three domains with different functions. The BH3 domain located at the N-terminus binds anti-apoptotic proteins such as BCL-XL and BCL-2. The central coiled-coil domain binds the *UVRAG* gene, which is associated with resistance to UV radiation and class III PI3K. The evolutionarily conserved domain binds class III PI3K and the membrane lipids of organelles. Beclin-1 also has a short C-terminal sequence that ensures an efficient nuclear export signal [58].

## SELECTIVE AUTOPHAGY - MITOPHAGY

Mitochondria are endosymbiotic organelles originating from primitive aerobic bacteria [59]. They are surrounded by a double membrane consisting of an outer mitochondrial membrane and an inner mitochondrial membrane [60]. Mitochondria are very dynamic organelles that constantly move and change their shape [61]. They are found in large numbers in most cell types and occupy about 10-40% of the cellular volume. The mitochondrial morphology and number depend on the cell type [60].

Mitochondria are important for the functioning and viability of the cell, and are essential in many processes, including energy production, metabolism and calcium buffering [61]. In eukaryotic cells, mitochondria are among the most important organelles, as they are involved not only in cellular energy generation, but also in cell differentiation, proliferation and apoptosis. The bioenergetic function of the mitochondria is oxidative phosphorylation. When electrons are transferred to complex I or complex II during this process, O_2_ is only partially reduced, which results in the formation of the superoxide anion, the precursor of most ROS. When ROS levels are too high, proteins, lipids and nucleic acids are oxidized [60]. Such oxidative stress, along with other adverse external conditions such as UV radiation or viral infections, can damage mitochondria, thus changing the mitochondrial permeability and stimulating apoptosis [62]. Aging and damaged mitochondria also generate reactive oxygen and nitrogen species themselves, and are characterized by reduced oxygen production, resulting in cell death, inflammation and aging. An increased number of abnormal organelles leads to the progression of many diseases, especially neurodegenerative diseases and cancer [59, 63].

Damaged mitochondria are removed from the cell by mitophagy, a selective type of autophagy in which the mitochondria are absorbed by the autophagosome, delivered to the lysosome and degraded by lysosomal enzymes. PTEN-induced putative kinase 1 (PINK1) and Parkin are the primary proteins involved in the process of mitophagy [64].

### The mechanism of mitophagy

**Figure 4:**
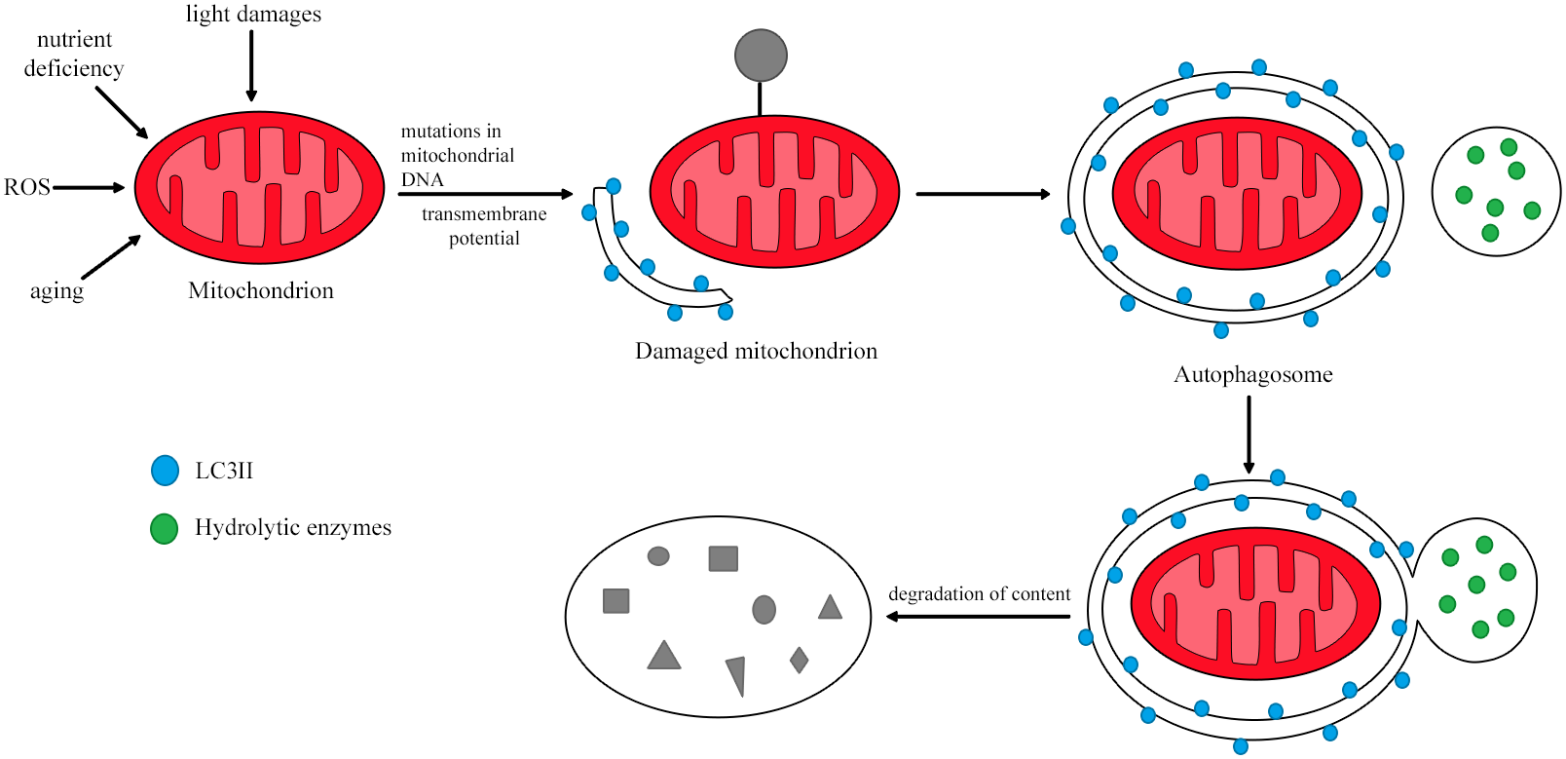
The process of mitophagy In response to stimuli such as nutrient deficiency, ROS and cellular aging, the mitochondrial membrane is depolarized. Damaged mitochondria are then removed through lysosomal degradation. Based on [64].

Mitophagy is the catabolic process that degrades dysfunctional mitochondria by directing the damaged organelles to lysosomes (Figure 4) [60, 65]. Thus, mitophagy maintains cellular homeostasis and is cytoprotective during disease development [66]. The regulation of mitochondrial morphological dynamics is strongly integrated with the initiation of mitophagy [67]. Mitochondria are cleaved by dynamin-related protein 1, while fusion of mitochondrial membranes involves three GTPases: mitofusins 1 and 2, which participate in external membrane fusion, and mitochondrial dynamin-like GTPase, which participates in internal membrane fusion. After the mitochondria have been divided, small polar and non-polar organelles are formed. The polarized ones can be fused, while the non-polarized ones are directed to the mitophagy pathway. Therefore, a reduced level of mitophagy has been observed in cases of cleavage inhibition and enhanced mitochondrial membrane fusion. On the other hand, the induction of fission promotes mitophagy [60].

Induction and substrate recognition are two important steps in mitophagy. In the first stage, initiated by ROS (generated by damaged mitochondria), proteins involved in this process inhibit mTOR and activate AMP kinase (AMPK) as a result of ATP reduction. This signal transduction involves both PI3K/AKT-dependent and -independent pathways. The PI3K/AKT-dependent pathway is a classic mTOR pathway involved in autophagy. Protein tyrosine kinase (PTK) and G protein-coupled receptors located on the cell surface, in combination with ligands present on the outside of the cell, phosphorylate phosphatidylinositol 4, 5-bisphosphate (PIP2), thus converting it to phosphatidylinositol 3, 4, 5-trisphosphate (PIP3). PIP3 is an important signaling molecule due its ability to combine with many proteins, including phosphoinositide-dependent protein kinase 1 and AKT. Phosphoinositide-dependent protein kinase 1 binds to PIP3 in the cell membrane, phosphorylating AKT. In turn, activated AKT phosphorylates various target proteins in damaged mitochondria, inhibiting the activation of mTOR complex 1 and then inducing mitophagy. The PI3K/AKT-independent pathway is activated by reduced mitochondrial ATP levels, which can activate liver kinase B1 or AMPK, thus inhibiting mTOR complex 1 activity. Activated AMPK phosphorylates tuberous sclerosis complex 2, increases GTPase activating protein activity, and converts Rheb-GTP into the Rheb-GDP homologue that participates in mitophagy. PINK1 and Parkin are involved in the second stage of mitophagy [62].

### The participation of PINK1 in mitophagy

PINK1 is a 63-kDa serine-threonine kinase consisting of 581 amino acids [68, 69]. The mitochondrial presenilin-associated rhomboid-like protease cleaves the PINK1 protein in the inner mitochondrial membrane into two smaller isoforms of 55 kDa and 45 kDa [68, 70]. PINK1 contains an N-terminal mitochondrial targeting sequence that allows it to bind to other proteins, and a C-terminal domain that regulates its autophosphorylation. PINK1 is mostly located near the inner mitochondrial membrane [69, 71]. PINK1 is a neuroprotective protein that prevents mitochondrial dysfunction and apoptotic cell death in response to stress conditions. Its activity, conducive to cell survival, is activated by several mechanisms, including phosphorylation of the mitochondrial proteins TRAP1 and Omi/HtrA2 [68, 70].

Undamaged polarized mitochondria are characterized by low levels of PINK1, and this prevents the mitophagy of healthy mitochondria. In normal mitochondria, PINK1 is imported into the intermembrane space, where PINK1 is degraded by presenilin-associated rhomboid-like proteases and the proteasome, such that a constant, low level of PINK1 is maintained [60, 72]. However, during depolarization, mitochondrial import is inactivated and proteasomal degradation occurs, so the concentration of PINK1 increases, indicating that mitochondrial damage has occurred [73]. During mitochondrial damage, as a result of depolarization, PINK1 accumulates on the outer mitochondrial membrane. There, it recruits Parkin (E3 ubiquitin ligase) from the cytosol to the surface of the mitochondria, which is a signal to induce mitophagy [60, 61, 72].

The translocation of Parkin into the mitochondria is important. PINK1 in the outer mitochondrial membrane phosphorylates serine and threonine resides of mitofusin 2, which is a signal for Parkin recruitment. Parkin ubiquitinates numerous proteins present in the outer mitochondrial membrane, which leads to the recruitment of various autophagy receptors (e.g., p62/SQSTM1, OPTN and NBR1). These receptors contain the LC-interacting region motif, which allows them to bind to the autophagosome. In the next stage, Parkin binds to AMBRA1 (a protein that promotes autophagy) in the outer mitochondrial membrane. This binding, in response to mitochondrial depolarization, stimulates autophagosome formation and thus induces LC3-II-dependent autophagy [60, 74].

### Mitophagy and PINK1 in cancer

Mitophagy is a multi-step, dynamic process involved in the development of cancer. In theory, mitophagy could inhibit or induce tumor growth, but the studies carried out so far suggest that increased mitophagy promotes the development of cancer. The process of mitophagy is initiated primarily by oxidative stress and DNA damage, which lead to genomic instability [60, 62]. Lack of nutrients and oxygen create the perfect microenvironment for tumor development. Autophagy caused by oxygen deficiency enhances cell survival, thus promoting tumor progression. In addition, autophagy induces resistance to chemotherapy [62, 75].

PINK1 plays a dual role in tumorogenesis, it can both stimulate and inhibit cancer. On the other hand, mitophagy is thought to protect cells from cancer development to a small extent. As mitochondria are the main organelles responsible for energy production, their dysfunction can promote the migration and spread of cancer cells. Under certain conditions, mitochondria may produce more peroxide ions than usual, which contributes to the formation of metastases [62]. When damaged mitochondria produce ROS that can damage DNA and induce tumor growth, mitophagy removes these dysfunctional organelles, preventing cancer from developing. Thus, mitophagy may have cytoprotective functions [60, 62]. Moreover, PINK1, which is associated with PTEN, is significantly involved in the development of cancer. *PTEN* is a tumor suppressor gene encoding a multifunctional phosphatase that inhibits the PI3K/AKT pathway, and mutations of this gene have been found in many types of cancer. Given its ability to inhibit the PI3K/AKT signaling pathway and to stimulate PINK1, PTEN is believed to prevent cancer progression [67]. In addition, PINK1 is considered to be a positive regulator of the cell cycle, and thus may induce tumor development [76].

Other proteins linking mitophagy with the development of cancer are Parkin, BCL2/adenovirus E1B 19-kDa interacting protein 3 (BNIP3) and AMBRA1. It is believed that Parkin may be a tumor suppressor, as common mutations in the *Parkin* gene deregulate the cell cycle [60]. The mechanisms by which Parkin suppresses tumor development are not fully understood. It is known, however, that Parkin moves to the mitochondria when the membrane potential is reduced, which leads to the ubiquitination of mitochondrial proteins and the recruitment of p62-LC3 and autophagosomes to the mitochondria. Parkin recruitment also inhibit BNIP3, a protein that links apoptosis with mitophagy in cancer. BNIP3 inhibits proteins belonging to the BCL-2 family, thereby activating apoptosis. BNIP3 simultaneously increases mitophagy by binding to autophagosomes via the LC3 region [75]. BNIP3 protein expression is often altered in cancer. High BNIP3 activity has been correlated with metastases of breast cancer and colon cancer, while the silencing of *BNIP3* may stimulate leukemia, pancreatic, colon and stomach cancer. Loss of BNIP3 activity prevents mitophagy and increases ROS production. On the other hand, high expression of AMBRA1 correlates with a worse prognosis in pancreatic cancer. Therefore, the *in vivo* studies carried out so far have demonstrated conflicting effects of the receptors and regulators of mitophagy on the development of cancer [60, 75].

Until now, conventional cancer treatment has mainly included surgery supplemented with chemotherapy or radiotherapy; however, resistance to treatment often occurs, and surgery is not always effective. The regulation of mitophagy has been applied in the treatment of cancer, and the most effective method has been to stimulate mitophagy. The combination of mitophagy inducers with radiotherapy and chemotherapy has enhanced the effectiveness of the current treatments. In part, mitophagy is thought to exert anti-cancer activity due to its involvement in the immune response. Further research is needed to better understand the mechanism of mitophagy so that it can be used in cancer therapy [62].

Given its key functions in mitophagy, PINK1 is a possible target for the treatment of cancer. PINK1 was also recently discovered to regulate the cell cycle, which underlines its potential as an anti-cancer therapeutic focus. The reduction of PINK1 activity limits the proliferation of tumor cells by inhibiting the cell cycle just before cell division. However, the inability of cells to divide due to PINK1 deficiency can induce chromosomal aberrations, genetic instability and aneuploidy, which can lead to the progression of many types of cancer [76, 77].

## CHAPERONE-MEDIATED AUTOPHAGY (CMA)

**Figure 5:**
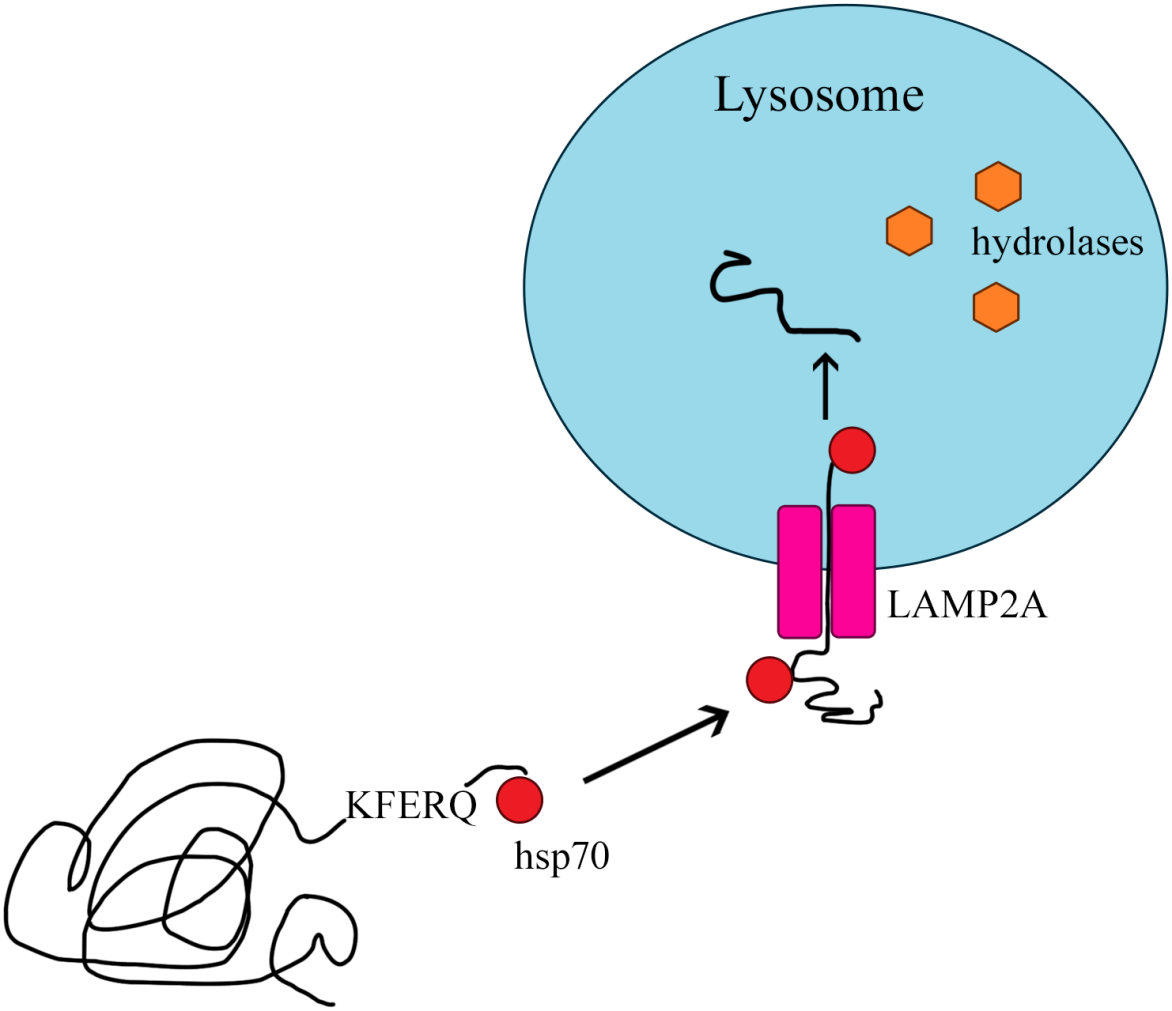
Chaperone-mediated autophagy

The delivery of proteins to the lysosome for degradation can take place in various ways. Not every type of autophagy involves the formation of lysosomal vesicles, as in the case of macroautophagy. Proteins can move from the cytosol to the lysosomal membrane and then pass through it into the lysosome [78] in the process of CMA. CMA was previously identified only in mammals [79, 80], and differs from the other types of autophagy in two basic ways [14, 81]: it is selective for the pool of cytosolic proteins, and it directs its substrate proteins to the lysosome one by one so that they can pass through the lysosomal membrane into its interior [79]. Like macroautophagy, CMA removes damaged and improperly folded proteins from the cell [12, 82]. The main proteins involved in this process are heat shock protein 70 (HSC70) and the lysosomal receptor LAMP2A (Figure 5) [83].

### Mechanism and regulation of CMA

Only proteins with a KFERQ pentapeptide motif are substrates for CMA [84]. The motif always consists of the same amino acids: a glutamine residue (Q); lysine (K); arginine (R) present at the beginning or end of the sequence; one of four hydrophobic amino acids (phenylalanine [F], valine, leucine or isoleucine); glutamic acid [E] or aspartic acid [14, 78]. Approximately 30% of cytosolic proteins are labeled with the KFERQ sequence [14]. The process of CMA involves four stages: substrate recognition and lysosomal targeting, substrate binding, substrate translocation, and substrate degradation within the lysosome [78].

Substrates are selectively recognized in the cytosol by a constitutive chaperone, HSC70, which delivers them to the lysosomal membrane [85]. This chaperone binds to the pentapeptide motif present in all CMA substrates. Then, the substrate-chaperone complex moves to the surface of the lysosome, where it is bound by the monomeric LAMP2A transmembrane receptor in the lysosomal membrane [78, 80, 86]. Thus far, LAMP2A is the only substrate-binding protein to be identified [87].

After the substrate protein binds to the LAMP2A monomer, LAMP2A multimerizes, forming a complex necessary to translocate the protein into the lysosome. During multimerization, LAMP2A receptor stability is maintained by HSC90, a protein on the inner side of the lysosomal membrane [12, 78]. The transport of the substrate requires ATP as an energy source and depends on the temperature (binding may occur even at 10°C, but transport is detectable only above 25°C) [14, 88]. Inside the lysosome, the substrate is hydrolyzed by proteolytic lysosomal enzymes [12, 78], and the molecules bound to HSC70 actively break down multimeric form LAMP2A. After being rapidly detached from the translocation complex, LAMP2A returns to its monomeric form and can bind further substrates, thus initiating a new cycle of binding and translocation [78, 88, 89].

The binding and translocation of the substrate protein are the most frequently coordinated steps of the CMA pathway, but can also occur separately. Therefore, the rate of CMA can be altered according to the speed of assembly/disassembly of the translocation complex. Many factors influence this process, including changes in the fluidity of the lysosomal membranes and the density of proteins in these membranes [78]. Moreover, glial fibrillary acid protein (GFAP) and elongation factor 1-alpha (EF1α) regulate the translocation complex. GFAP can exist in two forms: a non-phosphorylated variant that binds to and stabilizes LAMP2A in a multimeric complex, and a phosphorylated variant (GFAP-P) that binds to the lysosomal membrane outside this complex. Non-phosphorylated GFAP usually has a higher affinity for GFAP-P than LAMP2A, but the dimerization of GFAP-GFAP-P is often difficult due to the association of EF1α with GFAP-P. In the presence of GTP, EF1α is released from the lysosomal membrane, allowing the formation of the GFAP-GFAP-P dimer. Changes in the levels of GFAP-GFAP-P and EF1α in the lysosomal membrane, along with changes in the intracellular GTP and Ca^2+^ levels within lysosomes, activate CMA [80, 86, 88].

CMA is often activated during adverse conditions, such as nutrient deficiency (lack of nutrients above 10 hours), exposure to toxic components and oxidative stress, which are associated with elevated levels of HSC70 in the lysosome and LAMP2A in the lysosomal membrane. In fact, the level of LAMP2A in the lysosomal membrane directly determines CMA activity, since substrate interactions with LAMP2A are a limiting factor in this pathway [78, 88, 89, 90]. During mild oxidative stress, *de novo*-synthesized LAMP2A is delivered to the lysosome to increase CMA activity [88, 91]. Likewise, during nutrient deprivation, the level of LAMP2A increases, thus increasing the speed of CMA [91]. Interestingly, *de novo* synthesis of LAMP2A is not required under these conditions; rather, the LAMP2A complex is degraded and its constituent proteins are transferred from the lysosome to the lysosomal membrane [90]. The stability of HSC70 depends on the pH of the lysosomes, and a small increase in pH promots its degradation [78].

CMA activity declines with physiological aging, due to the reduced stability of LAMP2A in the lysosomal membrane. In aging animals, attempts were made to restore the normal CMA receptor level through genetic modification. These efforts significantly reduced the intracellular accumulation of oxidized proteins in aging tissues [91].

### CMA and carcinogenesis

Disorders of CMA occur in various pathological states, including cancers [78]. One of the most common CMA anomalies is dysfunction of the translocation complex. CMA activity and LAMP2A levels were reported to be greater in cancer cells than in the normal cells from which the tumor originated [79, 80, 89]. The mechanism responsible for the induction of CMA in tumors is still unknown, but it is postulated that the deregulation of microRNAs may underlie the increased expression of LAMP2A in cancer [78].

CMA, like macroautophagy, has two important effects on the development of cancer. On the one hand, it is a response to adverse conditions, protecting cancer cells against nutrient deficiency and thus enhancing their survival and proliferation. On the other hand, CMA removes tumor cells by inducing non-apoptotic/apoptotic cell death, thereby inhibiting cancer growth. In addition, CMA exerts anti-tumor activity in non-proliferative cancer cells by reducing mutant p53 protein levels via lysosomal degradation. CMA also promotes the proteolysis of other pro-oncogenic proteins in solid tumors, such as epidermal growth factor receptor pathway substrate 8 [79]. Autophagy disorders contribute to DNA damage, leading to genomic instability in some types of cancer [92]. It is believed that CMA has a pro-oncogenic function in cancer cells, while in normal cells, it has the opposite effect, protecting cells from intracellular and extracellular damage that could contribute to oncogenesis [78].

The relationship between CMA and glucose metabolism is worth noting [93]. When CMA activity increases, glycolysis must be maintained at a higher level to satisfy the bioenergetic demands of growing and proliferating cancer cells [79, 80]. Selective blockage of autophagy inhibits the transcription of many glycolytic enzymes, thus reducing glycolytic activity and reducing ATP production, which causes promoting tumor progression. However, in some cancers, the rate of glycolysis can be reduced by changes at the protein level. One of the most important enzymes limiting the rate of glycolysis is pyruvate kinase M2. The inhibition of CMA in cancer cells leads to the accumulation of an inactive form of pyruvate kinase M2 and thus reduces glycolytic activity [79, 89].

In the case of cancer, the adverse effects for patients following the inhibition of CMA result from limited quality control. When CMA is inhibited, ubiquitin-proteasome system activity increases, preventing the accumulation of damaged substrates that would usually be degraded by CMA. It is necessary to conduct further studies on CMA and cancer biology [80]. From a therapeutic point of view, CMA is a very promising treatment target, because its inhibition in murine tumors (and the accompanying reduction in LAMP2A activity) has been shown to effectively reduce tumor growth and metastasis [79, 89].

## MICROAUTOPHAGY

Microautophagy is the least characterized type of autophagy so far. It is a non-selective process in which proteins destined for degradation are transferred into the lysosome by being bent into its membrane, without the participation of the autophagosome. Small molecules are substrates for microautophagy. However, the exact mechanism of this process is not yet known [13].

## AUTOPHAGY AND THE IMMUNE SYSTEM

Autophagy has important functions in the immune system, as it directly eliminates pathogens, activates the inflammatory process, enables antigen presentation during infection and promotes the secretion of proinflammatory cytokines. Autophagy is considered the most primary form of innate resistance against microorganisms, as autophagosomes can directly capture and eliminate intracellular pathogens. Viruses and bacteria entering the body induce autophagy by competing for nutrients or stimulating innate immune receptors such as the toll-like receptors (TLRs). In the process of LC3-associated phagocytosis, microorganisms are captured by phagocytosis and remain in the intact vacuole, eventually forming autophagolysosomes that mature into autolysosomes. LC3-associated phagocytosis involves the Beclin-1-hVPS34 and LC3 complex, but is independent of ULK1 (a key initiator of autophagy), because phagocytosis is used to generate autophagosomes from the endoplasmic reticular internal membrane during nutrient deficiency [94, 95].

The binding of pathogen-associated molecular patterns with the appropriate receptors stimulates autophagy. The binding of HMGB1 and S100 to the advanced glycosylation end product-specific receptor can inhibit the phosphorylation of mTOR (a negative regulator of autophagy) or activate AMPK, which in turn inhibits mTOR and activates ULK1. These proteins also induce autophagy by their dissociation from the BCL-2 and BCL-XL complex, and through the MYD88 signaling pathway and the TRIF protein. Extracellular ATP and double-stranded DNA activate the inflammasome, which stimulates autophagosome formation through a signaling cascade including RABL, ULK1 and Beclin-1. Interferon (IFN)-γ and TNF-α induce autophagy via the ERK-dependent extracellular signaling pathway or IRG protein. Interleukin (IL)-1 and IFN-α/β also stimulate autophagy, but the mechanism of this process is not fully understood. On the other hand, IL-4, IL-10 and IL-13 suppress autophagy through the insulin receptor substrate 1 or PI3K pathway, activating mTOR [95, 96].

As Michael Lazarou explains, mitophagy is crucial for the immune system, as it prevents the release of mitochondrial DNA and ROS. By removing damaged mitochondria from the cell, autophagy limits the secretion of IL-1β and IL-18. Mitochondrial NLR family member X1 inhibits IFN production, while stimulating autophagy by interacting with the ATG5-ATG12 or ATG16L1 complex [95, 96].

Autophagy is involved in inflammatory diseases. The association of ATG16L1 and IRGM with Crohn's disease and a common form of intestinal inflammation has been established. In addition, autophagy provides cytosolic pathogen-associated molecular pattern molecules to TLRs (important regulators of the inflammatory process), which leads to the production of IFN-α by dendritic cells [94].

Autophagy also participates in the presentation of antigens (including viral and self-antigens) to CD4+ T cells. Antigen-presenting complexes capture extracellular antigens and deliver them to autophagosomes, where hydrolases generate immunogenic peptides that can be incorporated into Major Histocompatibility Complex II particles for presentation to CD4+ T lymphocytes. In the case of viral ligands, TLR7 and TLR9 stimulate autophagy, contributing to the production of IFN-α and improving antigen presentation [94, 95].

## CONCLUSIONS

Due to studies conducted over the last few years, the view on autophagy has changed significantly. Until recently, cell death was considered only in terms of apoptosis and necrosis. Autophagy is currently a very actively researched process.

Autophagy is important in the pathogenesis of many types of cancer. Its effects on cancer cells depend on many factors: the clinical stage of the tumor, the type of cancer, the cell environment and the physiological condition of the patient. In the development of cancer, autophagy has two contradictory functions. On the one hand, it can cause cell death, especially in the early stages of the disease. On the other hand, it causes the resistance of tumor cells to treatment, contributing to cancer progression and enabling cell survival, especially in the advanced stages of the disease (Table 2).

**Table 2:** Autophagy genes as tumor suppressors and promoters

Gene	Wild-type/mutant	Cancer type	Alteration in cancer	Effect of alteration on autophagy	References
BECN1	Mutant	Breast cancerOvarian cancerProstate cancer	Suppression	Inhibition	[55, 97, 98, 99]
BECN1	Mutant	LeukemiaLung cancerLiver cancer Endometrial cancer Colorectal cancer Glioblastoma Brain cancer	Activation	Inhibition	[58, 97]
ATG2B ATG9B ATG5 ATG12	Mutant	Colon cancer	Activation	Inhibition	[20]
ATG7	Wild-type	Lung cancer	Activation	Inhibition	[100]
DRAM1	Mutant	Melanoma	Activation	Inhibition	[101]
p53	Mutant	Many cancers	Activation	Inhibition	[99, 101]
p53	Wild-type	Many cancers	Suppression	Activation	[101]
LAMP2	Mutant	Pancreatic cancer	Suppression	Activation	[102]
LAMP2	Wild-type	Prostate cancer Thyroid cancer Colon cancer	Activation	Activation	[103]
LAMP3	Wild-type	Gastric and colorectal cancer	Activation	Activation	[38]
Parkin	Mutant	Ovarian cancer Breast cancer Bladder cancer Lung cancer	Activation	Inhibition	[104]

Autophagy may be the target anti-cancer treatments. Due to the dual role of autophagy in cancer development, the drugs used may inhibit or induce this process. For personalized treatments, molecular tests are needed to determine whether an inducer or inhibitor of autophagy should be used. Recent reports indicate that late-stage autophagy inhibitors such as CQ or HCQ can effectively inhibit autophagosome-lysosome fusion. In addition, the studies carried out so far have shown that the suppression of autophagy may enhance the effectiveness of currently used anti-cancer drugs. However, since the induction of autophagy promotes cell death and thus the elimination of cancer cells, both autophagy-inducing and autophagy-inhibiting strategies are extremely important for ongoing clinical trials.

Research on the molecular mechanisms underlying autophagy may contribute to the development of new methods of diagnosing and treating cancer and non-invasively detecting its early forms. The study of genes associated with autophagy and signaling pathways involved in the earliest stages of the disease may be important in the development of drugs that prevent the further progression of the disease.
